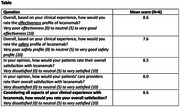# Physician Satisfaction With Lecanemab in Early Alzheimer's Disease: Real‐World Insights From Prescribers in the United States

**DOI:** 10.1002/alz70861_108605

**Published:** 2025-12-23

**Authors:** Gregory Cooper, Michael Henry Rosenbloom, Marwan N. Sabbagh, Jose Soria‐Lopez, Samuel Giles, Cara Leahy, Martin Sadowski, Curtis Schreiber, Paul E Schulz, David C Weisman, Christian J Camargo, Brooke Allen, Courtney Adams, Daryl Jones

**Affiliations:** ^1^ Norton Neuroscience Institute, Louisville, KY USA; ^2^ University of Washington Memory and Brain Wellness Center, Seattle, WA USA; ^3^ Barrow Neurological Institute, Phoenix, AZ USA; ^4^ The Neuron Clinic, San Diego, CA USA; ^5^ University of California San Diego, La Jolla, CA USA; ^6^ Memory Treatment Centers, Jacksonville Beach, FL USA; ^7^ Memorial Healthcare Institute for Neuroscience, Owosso, MI USA; ^8^ New York University Langone Health, New York, NY USA; ^9^ Missouri Memory Center, Citizens Memorial Hospital, Bolivar, MO USA; ^10^ John P. and Kathrine G. McGovern Medical School at UTHealth, Houston, TX USA; ^11^ Abington Neurologic Associates, Abington, PA USA; ^12^ University of Miami Miller School of Medicine, Miami, FL USA; ^13^ Roaring Fork Neurology, Basalt, CO USA; ^14^ Eisai Inc, Nutley, NJ USA; ^15^ Eisai Inc., Nutley, NJ USA

## Abstract

**Background:**

Lecanemab‐irmb (LEQEMBI®) is indicated for the treatment of patients with Alzheimer’s disease (AD) in the mild cognitive impairment or mild dementia stage. This analysis aimed to investigate neurologist’s satisfaction with lecanemab treatment in a real‐world patient population in the United States, in addition to patient and care partner satisfaction from their perspective.

**Method:**

This multicenter, retrospective case series and patient pathway study was conducted in 15 geographically diverse neurology clinics, each abstracting deidentified medical chart data for up to 25 patients receiving lecanemab (≥7 infusions) and 1 neurologist per site completing an electronic survey plus an interview. The electronic survey asked physicians to rate their satisfaction from very dissatisfied (0) to neutral (5) to very satisfied (10); the effectiveness and safety profile of lecanemab from very poor (0) to neutral (5) to very good (10). They were also asked to rate the patient and care partner satisfaction from their perspective. This is an interim analysis of the surveys completed to date (cutoff date: April 11, 2025). The protocol received central institutional review board exemption.

**Result:**

Responses from 6 neurologists averaged ratings of 8.6 on overall satisfaction with lecanemab, 8.6 on satisfaction with its effectiveness, 7.6 on satisfaction with its safety, 8.5 on patient satisfaction, and 8.0 on care providers' satisfaction with lecanemab (interim data). Satisfaction on clinical outcomes, including cognition, function, behavior, and quality of life were all rated ≥5 by the neurologist.

**Conclusion:**

Perception of lecanemab’s effectiveness and safety by neurologists was favorable, based on interim data. From the neurologist's perspective, both patients and their care partners were also satisfied with lecanemab. In the full data set analysis (data cutoff: May 23, 2025), perspectives on effectiveness and safety of lecanemab and satisfaction rates will be further explored.